# The effect of an antibiotic stewardship program on tigecycline use in a Tertiary Care Hospital, an intervention study

**DOI:** 10.1186/s12941-020-00377-9

**Published:** 2020-08-06

**Authors:** Rima Moghnieh, Dania Abdallah, Lyn Awad, Marwa Jadayel, Nicholas Haddad, Hani Tamim, Aline Zaiter, Diana-Caroline Awwad, Loubna Sinno, Salam El-Hassan, Rawad Lakkis, Rabab Khalil, Tamima Jisr

**Affiliations:** 1 Division of Infectious Diseases, Department of Internal Medicine, Makassed General Hospital, Beirut, Lebanon; 2 Division of Infectious Diseases, Department of Internal Medicine, Hôtel Dieu de France, Beirut, Lebanon; 3 Pharmacy Department, Makassed General Hospital, Beirut, Lebanon; 4 School of Pharmacy, Beirut Arab University, Beirut, Lebanon; 5 Infectious Disease and Residency Program, Internal Medicine, Central Michigan University, Saginaw, MI 48602 USA; 6 Department of Internal Medicine, American University of Beirut, Beirut, Lebanon; 7 Faculty of Medicine, Lebanese University, Beirut, Lebanon; 8 Department of Medical Research, Makassed General Hospital, Beirut, Lebanon; 9 Nursing Office, Makassed General Hospital, Beirut, Lebanon; 10 Faculty of Arts and Sciences, American University of Beirut, Beirut, Lebanon; 11 Department of Internal Medicine, Makassed General Hospital, Beirut, Lebanon; 12 Department of Laboratory Medicine, Makassed General Hospital, Beirut, Lebanon

**Keywords:** Tigecycline, Antibiotic stewardship, Off-label use, FDA-approved indication, Lebanon

## Abstract

**Background:**

A drug-oriented antibiotic stewardship intervention targeting tigecycline utilization was launched at Makassed General Hospital, Beirut, Lebanon, in 2016 as a part of a comprehensive Antibiotic Stewardship Program (ASP). In this study, we evaluated the effect of this intervention on changing tigecycline prescription behavior in different types of infections, patient outcome and mortality, along with tigecycline drug use density, when compared to an earlier period before the initiation of ASP.

**Methods:**

This is a retrospective chart review of all adult inpatients who received tigecycline for more than 72 h between Jan-2012 and Dec-2013 [period (P) 1 before ASP] and between Oct-2016 and Dec-2018 [period (P) 2 during ASP].

**Results:**

Tigecycline was administered to 153 patients during P1 and 116 patients during P2. The proportion of patients suffering from cancer, those requiring mechanical ventilation, and those with hemodynamic failure was significantly reduced between P1 and P2. The proportion of patients who received tigecycline for FDA-approved indications increased from 19% during P1 to 78% during P2 (*P *< 0.001). On the other hand, its use in off-label indications was restricted, including ventilator-associated pneumonia (26.1% in P1, 3.4% in P2, *P *< 0.001), hospital-acquired pneumonia (19.6% in P1, 5.2% in P2, *P *= 0.001), sepsis (9.2% in P1, 3% in P2, *P *= 0.028), and febrile neutropenia (15.7% in P1, 0.9% in P2, *P *< 0.001). The clinical success rate of tigecycline therapy showed an overall significant increase from 48.4% during P1 to 65.5% during P2 (*P *= 0.005) in the entire patient population. All-cause mortality in the tigecycline-treated patients decreased from 45.1% during P1 to 20.7% during P2 (*P *< 0.0001). In general, mean tigecycline consumption decreased by 55% between P1 and P2 (*P *< 0.0001).

**Conclusion:**

The drug-oriented ASP intervention targeting tigecycline prescriptions improved its use and patient outcomes, where it helped curb the over-optimistic use of this drug in off-label indications where it is not a suitable treatment option.

## Introduction

Tigecycline was first introduced to the Lebanese pharmaceutical market in 2006. It has demonstrated promising in vitro activity against antibiotic-resistant Gram-negative bacteria, including extended spectrum beta-lactamase-producing *Enterobacteriaceae*, carbapenem-resistant *Enterobacteriaceae*, and extensively drug-resistant (XDR) *Acinetobacter baumannii*. It is also active against various Gram-positive organisms, including *Staphylococcus aureus*, streptococci, and enterococci [1–3].

Multi-drug-resistant (MDR) and XDR *A. baumannii* has spread in Lebanese hospitals since 2004, becoming one of the primary nosocomial pathogens that compromises the outcome of hospitalized patients [4–8]. *A. baumannii* has displayed in vitro resistance to all available antimicrobial classes across different Lebanese hospitals, except for tigecycline and colistimethate sodium [4–8]. Therefore, physicians use tigecycline whenever a MDR or XDR pathogen is suspected or proven to cause a serious infection.

A utilization review was performed among inpatients that received tigecycline in our facility between 2012 and 2013 [9]. The tigecycline clinical success rate reached 43.4% and total mortality was 45% [9]. Stratifying tigecycline use among different patient subgroups revealed that it was mostly prescribed for indications not approved by the US Food and Drug Administration (FDA) or the European Medicines Agency (EMA) (81%), specifically in critically ill patients [9]. Total mortality was significantly higher in severely ill patients and for off-label indications, such as nosocomial pneumonia, bacteremia, and sepsis [9].

These results indicated a need to improve tigecycline prescription procedures. Therefore, a drug-oriented antibiotic stewardship intervention was launched in our facility in 2016 as a part of a comprehensive “Handshake Antibiotic Stewardship Program” (ASP) [10]. The primary endpoints of the current study were to observe the intervention effects on:

1. Shifting tigecycline use from the clinically vulnerable patient population toward the less critical population by avoiding its use in patients with signs of clinical severity, like hemodynamic failure and in those on mechanical ventilation.
2. Limiting tigecycline use to complicated intra-abdominal infections (cIAI) and to complicated skin and soft tissue infections (cSSTI), paired with reduced tigecycline prescription for infections like ventilator-associated pneumonia (VAP), hospital-acquired pneumonia (HAP), and bacteremia.
3. Changes in total tigecycline consumption and prescription trends resulting from ASP team oversight and controlling therapy duration, when possible, for patients who received it.

The secondary aims of this study were to assess patient outcome and all-cause mortality in patients who received tigecycline before and during the ASP. We also compared bacterial flora isolated from patients treated with tigecycline before and during the ASP.

## Methods

### Setting and study design

This was a retrospective chart review conducted at Makassed General Hospital, a 186-bed University hospital in Beirut, Lebanon. This study included adult inpatients who received tigecycline for more than 72 h between January 2012 and December 2013 [period 1 (P1), before ASP] and October 2016 to December 2018 [period 2 (P2), during ASP]. The hospital Institutional Review Board approved this study.

The ASP was adopted by the hospital starting in September 2016 and was based on the “handshake” strategy of prospective audit and immediate feedback to prescribers. The aims were to decrease high-end antibiotic use, namely antipseudomonal carbapenems and colistimethate sodium, and to control tigecycline use based on its utilization review [9, 10].

Regarding tigecycline intervention, a workshop was conducted in our facility to target broad-spectrum antibiotic prescribers, including infectious disease specialists, pulmonologists, and intensivists. This workshop discussed the results of the previous tigecycline utilization review and evaluated and agreed upon a new tigecycline use protocol. This protocol essentially limited tigecycline prescription to FDA/EMA-approved indications, namely cIAI and cSSTI.

The ASP team was given the authority to modify the choice and duration of the prescribed antimicrobial therapy after discussing the patient management plan and corresponding treatment guidelines with prescribers during daily clinical rounds.

### Data collection, definitions, and metrics for tigecycline use

The following information was collected during both periods: baseline demographic and clinical characteristics, indication for tigecycline therapy, tigecycline treatment strategy (empiric, targeted, monotherapy, or combination), duration of therapy, microbiological findings, clinical and microbiological outcomes, and all-cause mortality.

Monthly tigecycline use data were obtained from the hospital pharmacy. The World Health Organization (WHO) and Anatomical Therapeutic Chemical (ATC) classification systems were used to express data as WHO/ATC defined daily doses (DDD).

Tigecycline use density was measured as the number of DDD/1000 patient days (PD). The WHO definition was used to define tigecycline DDD as 0.1 g/day. The PD number was the number of patients present in any given location (e.g., hospital or ward) at a single time during a 24-h period [11]. We studied the quarterly change in tigecycline consumption levels and trends at P1 and P2.

Primary infections for which tigecycline was prescribed were defined according to clinical diagnostic criteria established by the U.S. Center for Disease Control and Prevention [12–14]. FDA- and EMA-approved indications for tigecycline were cSSTI and cIAI [15]. Off-label indications were HAP, VAP, urinary tract infection, diabetic ulcers, sepsis, bacteremia, and febrile neutropenia [15].

Empiric tigecycline use was defined as its administration to a patient with signs and symptoms of infection without a known bacterial isolate [9, 16]. Targeted therapy was defined as tigecycline administration in the presence of an identified organism [9, 16].

Clinical success was defined as an improvement in signs and symptoms of the primary infection treated by tigecycline, without the need to change the antibiotic regimen 72 h after starting tigecycline or without the need to restart other antibiotics within 72 h of discontinuing tigecycline [9, 17, 18]. The clinical success proportion was calculated as [9]:

$$({\text{Number}}\;{\text{of}}\;{\text{patients}}\;{\text{with}}\;{\text{clinical}}\;{\text{success}}/{\text{Total}}\;{\text{number of}}\;{\text{patients}}) \, \times \, 100.$$

Microbiological outcome success was defined as the eradication of the organism causing the primary infection during or after tigecycline therapy [9, 18]. Persistent identification of the same organism 72 h after initiating tigecycline therapy was considered a microbiological failure [9, 18]. The response was considered indeterminate when follow-up cultures were not available to verify eradication [9, 18]. The microbiological success proportion was calculated as [9]:

$$\left[ {{\text{Number}}\;{\text{of}}\;{\text{patients}}\;{\text{with}}\;{\text{microbiological}}\;{\text{success}}/({\text{Total}}\;{\text{number}}\;{\text{of}}\;{\text{patients }}{-}{\text{ Number}}\;{\text{of}}\;{\text{patients}}\;{\text{with}}\;{\text{undetermined}}\;{\text{microbiological}}\;{\text{response}})} \right] \, \times 100.$$

Mortality was quantified using 28-day all-cause mortality defined as deaths occurring between 72 h after treatment started and 28 days after tigecycline discontinuation [18].

Patient bacterial flora was assessed as a compilation of all bacterial isolates from any cultured site from patients treated with tigecycline. Bacterial identification was performed according to standard microbiological procedures. Antibiotic susceptibility was performed using the disc diffusion method as recommended by the Clinical and Laboratory Standards Institute (CLSI). All microbiological methods were consistent with CLSI guidelines for the corresponding year and antimicrobial susceptibility was determined using the CLSI breakpoints for the corresponding year [19]. The average turnaround time for bacterial identification and antibiogram results was 3 working days. Rapid diagnostic tests that detect antimicrobial resistance were not available in the hospital laboratory at the time of the study [20].

### Statistical analysis

Patients’ characteristics, treatment strategy, overall clinical outcome, microbiological outcome, all-cause mortality, and bacterial flora were quantified in patients during P1 and P2. The clinical outcome and all-cause mortality were also broken down by infectious disease diagnosis. The Statistical Package for Social Sciences program (SPSS Statistics for Windows, Version 23.0, IBM Corp., Armonk, NY, USA) was used for data entry, management, and analyses. Descriptive analysis was broken down by categorical independent variables for outcomes quantified using numbers and percentages. Bivariate analysis for ASP (P1 vs. P2) was conducted using the Chi square test for categorical variables and independent *t*-test for continuous variables. Parameters with P-value < 0.05 at the univariate level were considered statistically significant. The ASP impact on drug use density was evaluated using a segmented regression analysis of an interrupted time series adjusted for autocorrelation. We calculated the “change in level” as follows: [(mean P2 value – mean P1 value)/mean P1 value] × 100. We defined “change in trend” as the difference between the P1 and P2 change rates. The segmented regression analysis was applied using the newey command (considering Newey-West standard errors) in STATA version 15 (StataCorp LLC., College Station, TX). Statistical significance was defined as P-value< 0.05.

## Results

### Patient characteristics

Tigecycline was administered to 153 patients during P1 and 116 patients during P2. All patients’ demographic and clinical characteristics are detailed in Table 1. The comorbidities showed similar patterns during P1 and P2. However, the proportion of patients suffering from cancer decreased from 32.7% (50/153 patients) in P1 to 19% (22/116 patients) in P2 (*P *= 0.012). Similarly, the percentage of patients on mechanical ventilation dropped from 22.9% (35/153) during P1 to 11.2% during P2 (13/116) (*P *= 0.013), as well as those requiring vasopressor use [24.2% (37/153 patients) in P1 vs. 12.9% (15/116 patients) in P2, *P *= 0.021] (Table 1).

**Table 1:** Comparison of baseline demographic and clinical characteristics, indications, duration, and treatment strategy in patients who received tigecycline before and after the antibiotic stewardship program intervention

Characteristics	Patients treated with TGC before ASP (P1) (%) (N = 153)	Patients treated with TGC after ASP (P2) (%) (N = 116)	*P*
Age (years) (median; IQR)	68 (47–80)	70 (53–77)	0.314
≤ 65	72 (47.1%)	50 (43.1%)	0.519
> 65	81 (52.9%)	66 (56.9%)	
Gender			
Male	79 (51.6%)	64 (55.2%)	0.565
Female	74 (48.4%)	52 (44.8%)	
Comorbidities			
Cardiovascular disease	90 (58.8%)	83 (71.6%)	0.031
Diabetes	46 (30.1%)	48 (41.4%)	0.054
Respiratory disease	21 (13.7%)	19 (16.4%)	0.097
Acute renal failure	35 (22.9%)	10 (8.6%)	0.002
chronic kidney disease	19 (12.4%)	23 (19.8%)	0.097
Chronic hepatic disease	6 (3.9%)	2 (1.7%)	0.472
Neutropenia (ANC < 500 cells/µL)	30 (19.6%)	0	< 0.0001
Malignancy	50 (32.7%)	22 (19.0%)	0.012
Leukemia	24 (15.7%)	2 (1.7%)	< 0.0001
Lymphoma	11 (7.2%)	1 (0.9%)	0.013
Solid tumor	15 (9.8%)	19 (16.4%)	0.108
Bone marrow transplantation	14 (9.2%)	2 (1.7%)	0.011
Autologous	4 (2.6%)	1 (0.9%)	0.394
Allogeneic	10 (6.5%)	1 (0.9%)	0.026
Use of vasopressors before TGC use by 24 h	37 (24.2%)	15 (12.9%)	0.021
Mechanical ventilation	35 (22.9%)	13 (11.2%)	0.013
Indications for TGC therapy			
FDA approved	29 (19%)	91 (78.4%)	< 0.0001
Off-label	124 (81%)	25 (21.6%)	
FDA-approved indications			
cSSTI	18 (11.8%)	56 (48.3%)	< 0.0001
cIAI	4 (2.6%)	33 (28.3%)	< 0.0001
CAP	7 (4.6%)	2 (1.7%)	0.082
Off-label indications			
HAP	30 (19.6%)	6 (5.2%)	0.001
VAP	40 (26.1%)	4 (3.4%)	< 0.0001
BSI	14 (9.2%)	0	0.001
Sepsis	14 (9.2%)	3 (2.6%)	0.028
UTI	6 (3.9%)	2 (1.7%)	0.472
FN	24 (15.7%)	1 (0.9%)	< 0.0001
Diabetic ulcer	8 (5.2%)	8 (6.9%)	0.567
Duration of TGC therapy regardless the indication (days) (median/IQR)	8 (5–13)	7 (5–9)	0.973
≤ 7 days	67 (43.8%)	64 (55.2%)	0.064
> 10 days	55 (35.9%)	21 (18.1%)	0.001
> 15 days	27 (17.6%)	7 (6%)	0.005
Duration of TGC therapy in cIAI (days) (median/IQR)	10 (7–12)	7 (4–8)	0.137
> 5 days^a^	3/4 (75%)	22/33 (66.7%)	0.9
Duration of TGC therapy in cSSTI (days) (median/IQR)	8 (5–11)	7 (5–10)	0.499
> 7 days^b^	10/18 (55.6%)	26/56 (46.4%)	0.5
> 10 days^b^	5/18 (27.8%)	12/56 (21.4%)	0.75
Treatment strategy of TGC			
Empiric	67 (43.8%)	63 (54.3%)	0.087
Targeted	86 (56.2%)	53 (45.7%)	
Monotherapy	26 (17%)	47 (40.5%)	< 0.0001
Combination therapy^c^	127 (83%)	69 (59.5%)	

*ANC* absolute neutrophil count, *ASP* antibiotic stewardship program, *BSI* blood-stream infection, *CAP* community-acquired pneumonia, *cIAI* complicated intra-abdominal infections, *cSSTI* complicated skin and soft tissue infection, *d* days, *FN* febrile neutropenia, *HAP* hospital-acquired pneumonia, *IQR* interquartile range, *NA* not applicable, *P* period, *TGC* tigecycline, *UTI* urinary tract infection, *VAP* ventilator-associated pneumonia

^a^The denominator is the total number of cIAI cases per each category

^b^The denominator is the total number of cSSTI cases per each category

^c^Combination therapy for infections caused by Gram-negative bacteria

### Consumption of tigecycline

**Fig. 1:**
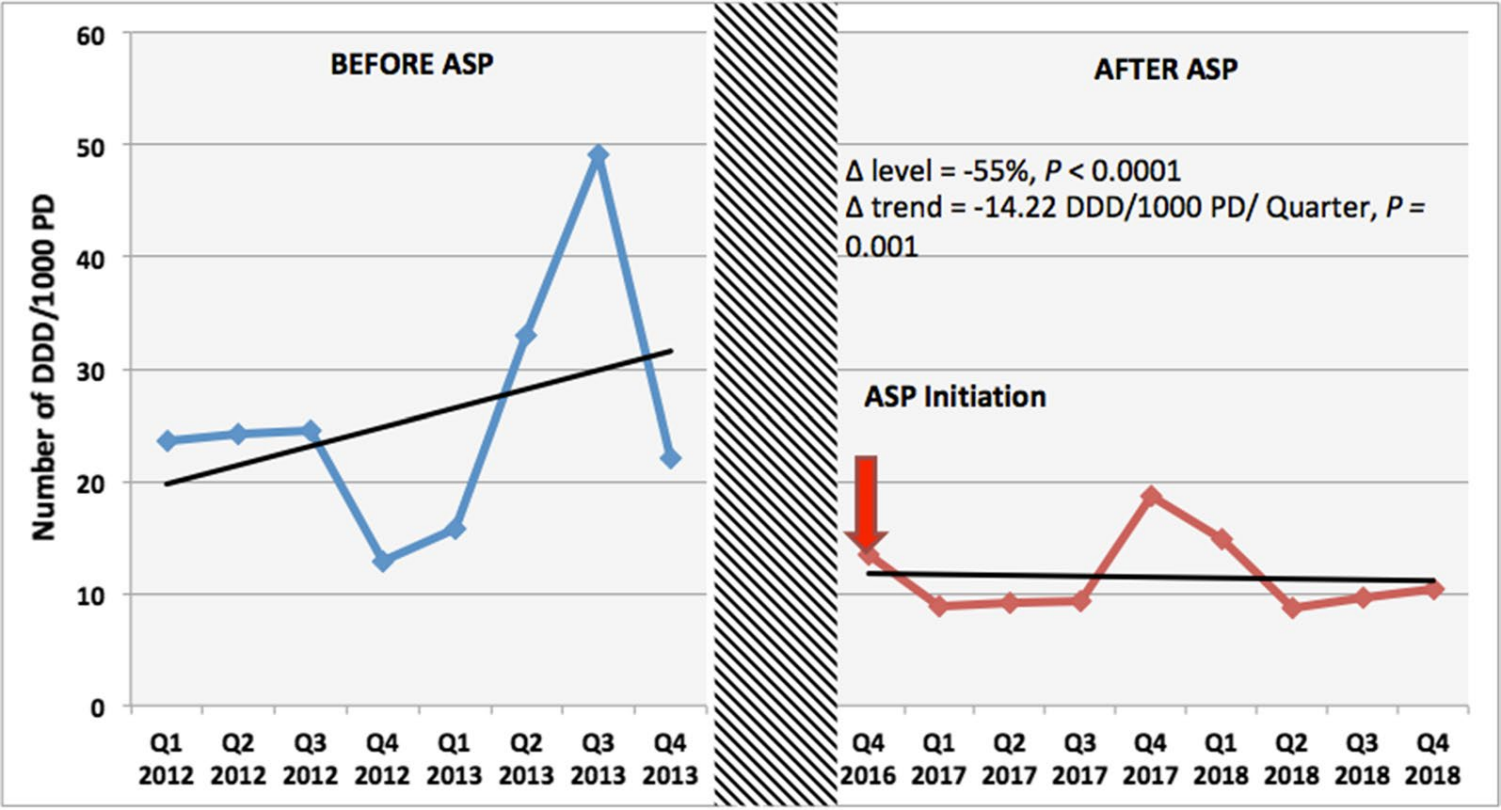
Quarterly variation of tigecycline consumption (DDD/1000 PD) before and after the antibiotic stewardship program implementation at Makassed General Hospital. *ASP* antibiotic stewardship program, *DDD* defined daily dose, *PD* patient days, *∆* change in

The average tigecycline consumption was 26 DDD/1000 PD during P1 compared to 11 DDD/1000 PD during P2; there was 55% decline in the drug density level (*P *< 0.0001). The ASP intervention resulted in a change in trend of − 14.22 DDD/1000 PD per quarter (*P *< 0.01) (Fig. 1).

### Indications of tigecycline

The proportion of patients who received tigecycline for cSSTI and cIAI, the FDA/EMA-approved indications, significantly increased between P1 and P2 (19% (29/153 patients) vs. 78% (91/116 patients) respectively, *P *< 0.001) (Table 1). Conversely, there was a significant decline in tigecycline prescription in all the observed off-label indications, including VAP [26.1% (40/153 patients) during P1 and 3.4% (4/116 patients) during P2, *P *< 0.001], HAP [19.6% (30/153 patients) during P1and 5.2% (6/116 patients) during P2, *P* = 0.001], BSI [9.2% (14/153 patients) during P1 and “Zero” during P2, *P* = 0.001], sepsis [9.2% (14/153 patients) during P1 and 3% (3/116 patients) during P2, *P* = 0.028] and febrile neutropenia [15.7% (24/153 patients) during P1 and 0.9% (1/116 patients) in P2, *P* < 0.001] (Table 1).

### Duration of therapy

The median duration of tigecycline therapy was 8 days (IQR, 5–13 days) during P1 and 7 days (IQR, 5–9 days) during P2 (*P *= 0.973), regardless of the indication (Table 1). In general, the intervention reduced the proportion of patients who received tigecycline for more than 10 days (35.9% during P1, 18.1% during P2, *P *= 0.001) and more than 15 days (17.6% during P1, 6% during P2, *P *= 0.005) (Table 1).

### Treatment strategy

Empiric use of tigecycline to treat primary infections increased from 43.8% (67/153 patients) during P1 to 54.3% (63/116 patients) during P2 (*P *= 0.087), whereas its use as a targeted therapy decreased from 56.2% (86/153 patients) during P1 to 45.7% (53/116 patients) during P2 (*P *= 0.087) (Table 1).

Tigecycline prescription for combination therapy with other antibiotics decreased from 83% (127/153 patients) during P1 to 59.6% (69/116 patients) during P2 (*P* < 0.0001), whereas it increased as a monotherapy from 17% (26/153 patients) during P1 to 40.5% during P2 (47/116 patients), (*P* < 0.0001) (Table 1).

### Patient outcome and mortality

The clinical success rate of tigecycline therapy showed an overall significant increase from 48.4% (74/153 patients) during P1 to 65.5% (76/116 patients) during P2 (*P *= 0.005) in the entire patient population (Table 2). Notably, individual clinical success rates for each indication were not significantly different between P1 and P2.

**Table 2:** Effect of the antibiotic stewardship program intervention on the clinical success, microbiological success and 28-day all cause mortality rates in patients treated with tigecycline in different subgroups

	Clinical Success			28-Day All Cause Mortality		
	Patients treated with TGC before ASP (P1) (%)	Patients treated with TGC after ASP (P2) (%)	*P*	Patients treated with TGC before ASP (P1) (%)	Patients treated with TGC after ASP (P2) (%)	*P*
Overall rate in all cases	74/153 (48.4%)	76/116 (65.5%)	0.005	69/153 (45.1%)	24/116 (20.7%)	< 0.0001
FDA approved indication	19/29 (65.5%)	67/91 (73.6%)	0.4	5/29 (17.2%)	15/91 (16.5%)	0.92
cSSTI	10/18 (55.5%)	44/56 (78.6%)	0.06	4/18 (22.2%)	9/56 (16.1%)	0.55
cIAI	3/4 (75%)	21/33 (63.6%)	1	0/4	6/33 (18.2%)	1
CAP	6/8 (75%)	2/2 (100%)	1	2/8 (25%)	0/2	0.43
Off-label indication	55/124 (44.4%)	9/25 (36%)	0.16	64/124 (51.6%)	9/25 (36%)	0.15
HAP	10/30 (33.3%)	0/6	0.56	22/30 (73.3%)	3/6 (50%)	0.34
VAP	12/40 (30%)	0/4	0.441	24/40 (60%)	1/4 (25%)	0.3
BSI	8/14 (57.1%)	0/0	NA	5/14 (35.7%)	0/0	NA
Sepsis	1/13 (7.7%)	0/3	1	12/13 (%)	3/3 (100%)	1
UTI	5/6 (83.3%)	1/2 (50%)	0.46	1/6 (16.7%)	0/2	1
FN	21/24 (87.5%)	1/1 (100%)	1	3/24 (12.5%)	0/1	1
Diabetic ulcer	5/8 (62.5%)	7/8 (87.5%)	0.57	1/8 (12.5%)	1/8 (12.5%)	1

The denominator in each case represents the total number of cases per category (first column). For example, the denominator for the clinical success rate for cSSTI during P1 (55.5%) represents the total number of cSSTI cases during P1 (n = 18)

*ASP* antibiotic stewardship program, *BSI* blood-stream infection, *CAP* community-acquired pneumonia, *cIAI* complicated intra-abdominal infections, *cSSTI* complicated skin and soft tissue infection, *FN* febrile neutropenia, HAP: hospital-acquired pneumonia, *NA* not applicable, *P* period, *TGC* tigecycline, *UTI* urinary tract infection, *VAP* ventilator-associated pneumonia

Follow-up cultures to assess the microbiological success or failure were only available for 39.2% of patients (60/153) in P1 and 22.4% of patients (26/116) in P2. During P1, the microbiological success rate was 43% (26/60 patients), compared to 19% (5/26 patients) during P2 (*P *= 0.03) (Table 2).

All-cause mortality in the entire tigecycline-treated patient population decreased from 45.1% (69/153 patients) during P1 to 20.7% (24/116 patients) during P2 (*P *< 0.0001) (Table 2). Mortality rates did not change based on the type of infection tigecycline was prescribed for.

### Microbiological flora

The microbiological culture results from patients treated with tigecycline during P1 and P2 were very similar, with few exceptions (Table 3). During both periods, the majority of cultured organisms were Gram-negative bacteria [81.3% of isolates (304/374) during P1 and 88.9% of isolates (296/333) during P2], with *A. baumannii* and *Enterobacteriaceae* predominating. The proportion of carbapenem-resistant *A. baumannii* from all isolated bacteria decreased from 23.3% (87/374 isolates) during P1 to 17.1% (57/333 isolates) during P2 (*P *= 0.04). *Enterobacteriaceae* species resistant to third generation cephalosporins constituted 17% of all isolated bacteria (64/374 isolates) during P1 compared to 22% (73/333 isolates) during P2. Specifically, *Klebsiella* spp. resistant to third generation cephalosporins represented 4.8% (18/374 isolates) of the isolated flora during P1 and increased to 9.3% (31/333 isolates) during P2 (*P *= 0.02) (Table 3). Carbapenem resistance among *Escherichia coli* and *Klebsiella* spp. emerged following the ASP [(0.6% (2/333 isolates) during P1 and 3.9% (13/333 isolates) during P2].

**Table 3:** Comparison of the bacterial flora isolated from patients treated with tigecycline before and after the antibiotic stewardship program intervention

Bacteria	Isolated organisms before ASP intervention (N = 374 organisms) (P1) (%)	Isolated organisms after ASP intervention (N = 333 organisms) (P2) (%)	*P*
Gram-negative species	304 (81.3%)	296 (88.9%)	0.005
*Escherichia coli*	60 (16%)	56 (18.8%)	0.78
3GC S	14 (3.7%)	14 (4.2%)	0.75
3GC R	46 (12.3%)	42 (12.6%)	0.89
CAR S	60 (16%)	54 (16.2%)	0.95
CAR R	0	2 (0.6%)	0.13
*Klebsiella species*	48 (12.8%)	44 (13.2%)	0.88
3GC S	30 (8%)	13 (3.9%)	0.02
3GC R	18 (4.8%)	31 (9.3%)	0.02
CAR S	48 (12.8%)	31 (9.3%)	0.14
CAR R	0	13 (3.9%)	< 0.0001
*Enterobacter species*	5 (1.3%)	11 (3.3%)	0.08
*Proteus mirabilis*	19 (5.1%)	14 (4.2%)	0.58
*Pseudomonas aeruginosa*	45 (12%)	50 (15%)	0.25
CAZ S	34 (9.1%)	36 (10.8%)	0.44
CAZ R	4 (1.1%)	14 (4.2%)	0.008
CAZ susceptibility NA	7 (1.9%)	0	–
CAR S	18 (4.8%)	32 (9.6%)	0.01
CAR R	20 (5.4%)	18 (5.4%)	0.97
CAR susceptibility NA	7 (1.9%)	0	–
*Acinetobacter baumannii*	105 (28.1%)	71 (21.3%)	0.04
CAR S	12 (3.2%)	14 (4.2%)	0.48
CAR R	87 (23.3%)	57 (17.1%)	0.04
CAR susceptibility NA	6 (1.6%)	0	–
*Stenotrophomonas maltophilia*	7 (1.9%)	18 (5.4%)	0.01
Gram-positive species	70 (18.7%)	37 (11.1%)	0.005
*Staphylococcus aureus*	11 (2.9%)	7 (2.1%)	0.48
MET S	5 (1.3%)	5 (1.5%)	0.76
MET R	6 (1.6%)	2 (0.6%)	0.22
*Enterococci*	19 (5.1%)	11 (3.3%)	0.24
VAN S	9 (2.4%)	8 (2.4%)	0.99
VAN R	0	3 (0.9%)	0.07

*ASP* antibiotic stewardship program, *CAR* carbapenem, *CAZ* ceftazidime, *MET* methicillin, *NA* not available, *P* period, *R* resistant, *S* susceptible, *VAN* vancomycin, *3GC* third generation cephalosporin

In cultured Gram-positive bacteria, methicillin-resistant *S. aureus* isolation decreased during the intervention period [1.6% of isolates (6/374) during P1 and 0.6% of isolates (2/333) during P2, *P *= 0.22]. Additionally, vancomycin-resistant *Enterococci* were isolated from patients at P2 (0.9%, 3/333 isolates) (Table 3).

## Discussion

This study observed the effects of an ASP for tigecycline use among inpatients by comparing the 2 years before the intervention to 2 years during the intervention. Before the intervention, a formulary restriction policy was used to control prescription of broad-spectrum antibiotics in our facility, including tigecycline. During the intervention period, a dedicated ASP team was implemented to prospectively audit prescribed antimicrobials and give immediate feedback during daily ward rounds. The program also included educational activities for prescribers regarding rational use of antibiotics and disseminating guidelines for the management of common infectious diseases in our facility.

Patients’ characteristics and comorbidities were similar before and during the intervention, including older age and the presence of comorbid illness like cardiovascular disease, diabetes, and respiratory disease. This demonstrates that a similar range and complexity of cases were being treated at our tertiary care facility during the study [*Internal Hospital Data*]. Yet, we observed a change in tigecycline use and therapy strategy, where tigecycline prescriptions to treat infections were reduced in neutropenic patients with cancer, patients on mechanical ventilation, and patients with hemodynamic failure.

In 2010 and again in 2013, the U.S. FDA issued a boxed warning regarding the increased risk of mortality with tigecycline therapy compared to alternatives for approved and unapproved indications, thus cautioning its administration for all cases and advising the use of available alternative antibiotics [21, 22].

At the time of the intervention, XDR *A. baumannii* and carbapenem-resistant *Enterobacteriaceae* were on the rise in most Lebanese hospitals and many prescribers were eager to avoid using carbapenem whenever possible [23–26]. The recently approved antibiotic formulations containing cephalosporins and beta-lactamase inhibitors that show promise against these organisms, such as ceftolozane/tazobatam and ceftazidime/avibactam, were not available in Lebanon during the study. Accordingly, tigecycline use continued during the ASP as part of the carbapenem-sparing strategy, but the intervention succeeded in decreasing tigecycline use in high-risk and immunocompromised patients.

The type of infection was also a consideration. Tigecycline was primarily restricted to FDA-approved indications per our ASP protocols. It was mainly prescribed to manage acute bacterial cSSTI and cIAI in non-critically ill patients. Its use in severely ill patients and for off-label indications like HAP, VAP, bacteremia, sepsis, and febrile neutropenia was significantly reduced. Shifting the types of infections treated with tigecycline was one of the main priorities of the ASP. The empiric use of tigecycline as a mono- or combination therapy to reduce carbapenem use in non-severely ill patients with cSSTI and cIAI who were at risk for infection with MDR bacteria was supported by national and international guidelines, multicenter studies, and expert opinions [16, 27–32].

The ASP significantly decreased tigecycline consumption levels by 55%, which was accompanied by a prominent reduction in its prescription rate. It is well known that unnecessary, increased antibiotic consumption is positively correlated with the emergence of antibiotic resistance [33–35]. Shifting tigecycline prescription from off-label to FDA-approved indications, in addition to shortening therapy duration under appropriate conditions based on international guidelines, collectively reduced its consumption and prescription rates.

The intervention produced a significant decline in tigecycline prescription for more than 10 days, with the mean therapy duration being 7 days (IQR, 5–9 days), regardless of the indication. An extended duration of antibiotic therapy is usually associated with emergence of resistance because selection of antibiotic-resistant strains increases over the time of antibiotic exposure [36]. The Infectious Disease Society of America (IDSA) and Society for Healthcare Epidemiology of America guidelines for ASP implementation in hospitals strongly recommend strategies that reduce antibiotic therapy to the shortest effective duration [37]. For instance, recently updated international guidelines indicate that the optimal duration of antibiotic therapy is 3–5 days in cIAI cases where patients undergo an adequate source-control procedure [28, 29]. The duration can be extended to 7 days depending on the presence of concomitant bacteremia, rate of fever resolution and other signs of infection, and the presence of comorbidities [27, 38]. For other infections like acute bacterial cSSTI, the latest IDSA guidelines suggest 7 to 10 days of therapy with individualization based on clinical response and factors like comorbidities, etiology, and appropriateness of drug or dosages [39].

ASPs aim to effectively control antibiotic utilization and antimicrobial resistance rates [37]. In Lebanon, carbapenem resistance has been on the rise over the past 10 years in clinically-relevant Gram-negative bacteria, an alarming situation in light of limited resources [23–26]. Colistin resistance has also recently been detected in *Enterobacteriaceae* and *Acinetobacter* species from clinical samples [40, 41].

One potential side effect of drug-oriented ASPs is that decreasing consumption of one antimicrobial can result in increased consumption of another one. Despite similar patient population complexity in our tertiary care facility before and during the intervention, decreased tigecycline use was not compensated for by increased consumption of carbapenems or colistin [10]. Implementing the handshake ASP in our facility led to an important reduction in the density and rate of prescribing other broad-spectrum antibiotics, including the antipseudomonal carbapenems imipenem and meropenem and colistimethate sodium [10]. Our ASP protocols were not only based on modifying antibiotics, but on stratifying patients according to the risk of infection or acquisition of Gram-negative organisms resistant to carbapenems and extended-spectrum cephalosporins [10]. This key measure allowed us to properly choose empiric treatment options, thus sparing empiric use of carbapenems and colistin when suitable. Antibiotic therapy was escalated or de-escalated to suit the patient’s condition when microbiological culture results were available.

Notably, there was a compensatory increase in consumption of other antibiotics, like piperacillin-tazobactam and third and fourth generation cephalosporins, in the hospital as a whole. This was due to the various complicated cases frequently managed in our facility, such as neutropenic patients with cancer, bone marrow transplant recipients, and critically ill patients [10]. Piperacillin-tazobactam is a less potent inducer of antimicrobial resistance in Gram-negative bacteria compared to carbapenems, fluoroquinolones, and extended-spectrum cephalosporins [42, 43].

Similar to our predictions, the clinical outcome of patients treated with tigecycline did not change during the ASP based on the type of infection only. However, the ASP improved patient outcomes across the entire tigecycline-treated cohort and decreased overall mortality. Favorable outcome rates and decreased death were due to prescribing tigecycline for FDA-approved indications and avoiding its use for off-label indications where it is not effective, such as HAP, VAP, bacteremia, and sepsis, and in cases with high mortality risks, as per the FDA boxed warning [21, 22].

We also observed the bacterial flora in patients who received tigecycline before and during the intervention, which does not represent the full hospital ecology. Microbiological culture results were similar during both periods, with mostly Gram-negative bacteria isolated from clinical samples. However, fewer patients with carbapenem-resistant *A. baumannii* received tigecycline during the intervention because most of the cases infected with this organism were critically ill and suffering from the off-label diseases HAP or VAP [*Internal Hospital Data*]. Conversely, we observed an increase in *Enterobacteriaceae* species resistant to third generation cephalosporins and the emergence of carbapenem resistance in these species. The ASP did not induce this issue because the rate of change of susceptibility patterns of nosocomial flora to tigecycline and other available antibiotics lags behind that of antibiotic prescription during the observed intervention period [44]. It may have been induced by the extensive use of carbapenems and other broad-spectrum antibiotics before the ASP. It is important to note that tigecycline does not stimulate cross-resistance to other antibiotic classes [15]. Its induction of resistance does not have the same impact on patient outcomes or altering the microbiome that is observed for other antibiotics after excessive use [45, 46].

## Limitations and strengths

A main limitation in this study is that it does not consider alternative antibiotics used for non-FDA-approved indications along with the corresponding patient outcome, particularly in the context of increasing carbapenem resistance. Its retrospective design, small sample size, and lack of adjusted analyses for results that are subject to confounds are also considerable limitations. The observed microbiological success rate was also subject to surveillance bias because most cases did not have follow-up cultures.

Nevertheless, this study highlights the importance of drug utilization reviews and how ASPs can reduce drawbacks when using newly introduced antibiotics. This study details a real-life experience in a developing country where the incidence of nosocomial extensively drug-resistant organisms has steadily increased, creating a significant threat of no antimicrobial options in the therapeutic armamentarium.

## Conclusion

The ASP targeting tigecycline prescriptions improved its use and patient outcomes. Tigecycline played an important role in managing cIAI and acute bacterial cSSTI during the antibiotic resistance era, when it was crucial to spare carbapenem use. Our targeted intervention helped to curb the over-optimistic use of this drug in off-label indications where it is not a suitable treatment option.

## Data Availability

The data that support the findings of this study are available from Makassed General Hospital but restrictions apply to the availability of these data, which were used under license for the current study, and so are not publicly available. Data are however available from the authors upon reasonable request and with permission of Makassed General Hospital.

## Abbreviations

ASP: Antibiotic stewardship program
ATC: Anatomical therapeutic chemical
cIAIs: Complicated intra-abdominal infections
CLSI: Clinical and Laboratory Standards Institute
cSSTIs: Complicated skin and soft tissue infections
DDD: Defined daily dose
EMA: European Medicines Agency
FDA: Food and Drug Administration
HAP: Hospital-acquired pneumonia
IDSA: Infectious Disease Society of America
MDR: Multi-drug-resistant
P1: Period 1
P2: Period 2
PD: Patient days
VAP: Ventilator-associated pneumonia
XDR: Extensive-drug-resistant
